# Long-Term Prognosis of Cognitive Function in Patients With Idiopathic Normal Pressure Hydrocephalus After Shunt Surgery

**DOI:** 10.3389/fnagi.2020.617150

**Published:** 2021-01-20

**Authors:** Akihiro Kambara, Yoshinaga Kajimoto, Ryokichi Yagi, Naokado Ikeda, Motomasa Furuse, Naosuke Nonoguchi, Shinji Kawabata, Toshihiko Kuroiwa, Kenji Kuroda, Shohei Tsuji, Ryuichi Saura, Masahiko Wanibuchi

**Affiliations:** ^1^Department of Neurosurgery, Osaka Medical College, Takatsuki, Japan; ^2^Department of Neurosurgery, Tesseikai Neurosurgery Hospital, Shijonawate, Japan; ^3^Clinical Department of Rehabilitation, Osaka Medical College, Takatsuki, Japan; ^4^Department of Physical and Rehabilitation Medicine, Osaka Medical College, Takatsuki, Japan

**Keywords:** hydrocephalus, cognition, dementia, shunting, long term outcome

## Abstract

The long-term prognosis of cognitive function in patients with idiopathic normal pressure hydrocephalus (iNPH) remains unclear. This study aimed to determine the long-term prognosis of cognitive function in patients with iNPH, as well as the factors related to it. It included 48 patients with iNPH who were treated with cerebrospinal fluid shunting between January 2015 and December 2017 at Osaka Medical College Hospital, with follow-up evaluation of their cognitive function for >2 years. Cognitive function was measured using the Mini-Mental State Examination (MMSE) preoperatively and at 3 months, 1 and 2 years post-operatively. The mean MMSE score (22.4 ± 5.4 preoperatively) improved at 3 months [23.8 ± 5.0 (*p* = 0.0002)] and 1 year [23.7 ± 4.8 (*p* = 0.004)] post-operatively. At 2 years post-operatively, they were able to maintain their preoperative level (22.6 ± 5.3). The patients were classified in to the cognitive decline group [11 (23%) patients; a decrease in the MMSE score by ≥ 2 points 2 years after surgery] and the maintenance/improvement group [37 (77%) patients]. Univariate and receiver operating characteristic analyses were performed for the two groups to identify factors associated with cognitive prognosis. In both groups, the patients who were younger (*p* = 0.009) or had milder symptoms (*p* = 0.035) had a better long-term prognosis of cognitive function. The cutoffs for age and disease severity (idiopathic normal-pressure hydrocephalus grading scale; INPHGS) were 78 years (area under the curve = 0.77) and 5 points (area under the curve = 0.71), respectively. In conclusion, most patients (77%) were able to improve and maintain cognitive function for at least 2 years after surgery. The fact that disease severity and age are associated with cognitive prognosis suggests that early iNPH intervention is desirable to improve cognitive prognosis.

## Introduction

Idiopathic normal pressure hydrocephalus (iNPH) is clinically characterized by gait disturbance, dementia, and urinary incontinence (Vanneste, 2000; Relkin et al., 2005). Cerebrospinal fluid (CSF) shunting can be used to treat it (Kuriyama et al., 2017). Currently, iNPH is considered a rare disease; however, several recent population-based epidemiological studies have shown that iNPH is common and affects 2–3% of older adults (Hiraoka et al., 2008; Tanaka et al., 2009; Andersson et al., 2019). Given that the progression of gait and cognitive impairment leads to the requirement of care at home and in the community, iNPH is becoming increasingly important with respect to public health and social security.

To reduce the caregiving burden on families and society, iNPH treatment for short- and long-term improvement and maintenance of gait and cognitive function is required. There have been numerous reports regarding the long-term prognosis of gait function after CSF shunt surgery in patients with iNPH. Gait function was found to be improved in ~80% of cases at 3 years post-operatively; moreover, the long-term prognosis of gait function was good (McGirt et al., 2008; Pujari et al., 2008).

However, few studies have reported the long-term prognosis of cognitive function in patients with iNPH, with most studies having a follow-up period of about 1 year (Yamada et al., 2017). Even in the reports of long-term prognosis, there are no studies reporting the post-operative progression as measured using cognitive function tests, and the prognostic factors have not been clearly reported (Koivisto et al., 2016; Grasso et al., 2019). Consequently, the long-term cognitive prognosis, course of iNPH after 2 post-operative years, and their related factors remain unclear. This retrospective study aimed to determine the long-term cognitive trends and factors that affect long-term cognitive function in patients with iNPH after CSF shunt surgery.

## Materials and Methods

### Eligible Patients

Out of the 72 patients with iNPH who were treated with CSF shunting from January 2015 to December 2017 at Osaka Medical College Hospital, we included 48 patients whose cognitive function could be evaluated with follow-up for > 2 years.

The excluded cases included 10 deaths (6, 1, 1, 1, and 1 cases of pneumonia, bladder cancer, head injury, subarachnoid hemorrhage, and sudden death of unknown cause, respectively), 4 transfers, 10 cases of distance-related difficulties in accessing the hospital, and 1 case of difficulty in completing the Mini-Mental State Examination (MMSE).

The indications for surgery were according to the Japanese Idiopathic Normal Pressure Hydrocephalus Treatment Guidelines, 3rd edition. All patients with ≥1 gait, cognitive, or urinary deficit who presented with enlarged ventricles (Evans index >0.3) and lacked other neurological or non-neurological diseases that explained the aforementioned clinical symptoms or a previous history of any disease that could cause enlarged ventricles were enrolled (Relkin et al., 2005; Jaraj et al., 2017). CSF shunting was performed in patients with a positive CSF tap test and in those with a negative tap test but with disproportionately enlarged subarachnoid space hydrocephalus on magnetic resonance imaging (Mori et al., 2012).

### Measurement Parameters

We retrospectively obtained the following measurement parameters: age, sex, idiopathic normal pressure hydrocephalus grading scale (INPHGS) (Mori, 2001), comorbidities, time to surgery, and surgical technique. A speech therapist administered the MMSE (Folstein et al., 1975) to the patients before the spinal tap test and at 3 months, 1 and 2 years after the CSF shunt surgery. The MMSE is useful as a screening tool for dementia and gives information about the severity of dementia.

The INPHGS consists of sections on gait disturbance, dementia, and urinary incontinence. Gait disturbance is defined as 0, normal; 1, unstable but independent gait; 2, walking with one cane; 3, walking with two canes or a walker frame; and 4, walking not possible. Dementia is defined as 0, within the normal range; 1, no apparent dementia but apathetic; 2, socially dependent but independent at home; 3, partially dependent at home; 4, totally dependent. Urinary incontinence is defined as 0, absent; 1, absent but with pollakiuria or urinary urgency; 2, present sometimes only at night; 3, present sometimes even during the day; 4, frequent. The grades of gait disturbance, dementia, and urinary incontinence were summated to obtain the total grade, which ranges from 0 to 12.

To investigate the factors associated with poor cognitive prognosis, the patients were divided into two groups: the maintenance/improvement group (*n* = 37) and the decline group (*n* = 11). A previous study has reported that a mean difference of 1.1 points was observed when the MMSE was administered twice, 24 h apart, by the same tester (Mori et al., 2012). Therefore, a one-point reduction was considered to be within the error range and the group that dropped more than 2 points was considered to be the decline group.

### Statistical Analysis

Changes in the MMSE score were assessed using Wilcoxon's signed-rank test. Between-group differences in age, sex, comorbidities, time to surgery, operative technique, and disease severity were assessed using Pearson's chi-square test and Student's *t-*test. Parameters significantly associated with long-term cognitive function were entered into a logistic regression model to assess the cutoff values and area under the curve. Receiver operating characteristic curves were drawn to visualize the effect of these parameters on cognitive prognosis.

Moreover, factors associated with the prognosis of cognitive function were extracted using a decision tree analysis.

Data analyses were performed using JMP Pro 15.1. This software was developed by SAS in North Carolina, USA, in 1989 to help researchers and technicians visually analyze data.

## Results

### Demographic and Clinical Data

Table 1 lists the patients' baseline characteristics. Their mean age was 76.9 (±5.8) years; there were 29 (60.4%) men and 19 (39.6%) women. The mean INPHGS score was 5.3 (±2.3) points. The average number of days from the tap test to surgery was 62.9 (±30) days. The surgical form was lumboperitoneal and ventriculoperitoneal shunting in 55.1 and 43.2% of patients, respectively. There were 43 (89.6%) patients who were administered the INPHGS; further, CSF shunting improved iNPH symptoms in most patients. In terms of major comorbidities, 6 patients had Alzheimer's disease (AD), 13 had hypertension, 12 had diabetes, 11 had hyperlipidemia, and 6 had cerebral infarction.

**Table 1:** Demographic and clinical characteristics of the patients (*n* = 48).

**Clinical characteristic (*n* = 48)**	
Age, mean (SD)	76.9 (±5.8)
**Sex, % (n)**	
Men	60.4% (29)
Women	39.6% (19)
INPHGS, mean (SD)	5.3 (±2.3)
MMSE score, mean (SD)	22.4 (±5.4)
Days until surgery, mean (SD)	62.9 (±30.)
**Type of surgery, % (n)**	
LP shunt	81.2% (39)
VP shunt	18.8% (9)
**INPHGS, % (n)**	
Responder	89.6% (43)
Non-responder	10.4% (5)

*The total scores of gait disturbance, dementia, and urinary incontinence were evaluated on a 12-point scale. Improvement (responder) was considered as a decrease in the total INPHGS score by least one point at 1 year after surgery, when the average MMSE score peaked, compared to the preoperative levels*.

*INPHGS, Idiopathic Normal Pressure Hydrocephalus Grading Scale; MMSE, Mini-Mental State Examination; LP, Lumboperitoneal; VP, Ventriculoperitoneal; SD, Standard deviation*.

### Changes in MMSE Over Time

**Figure 1:**
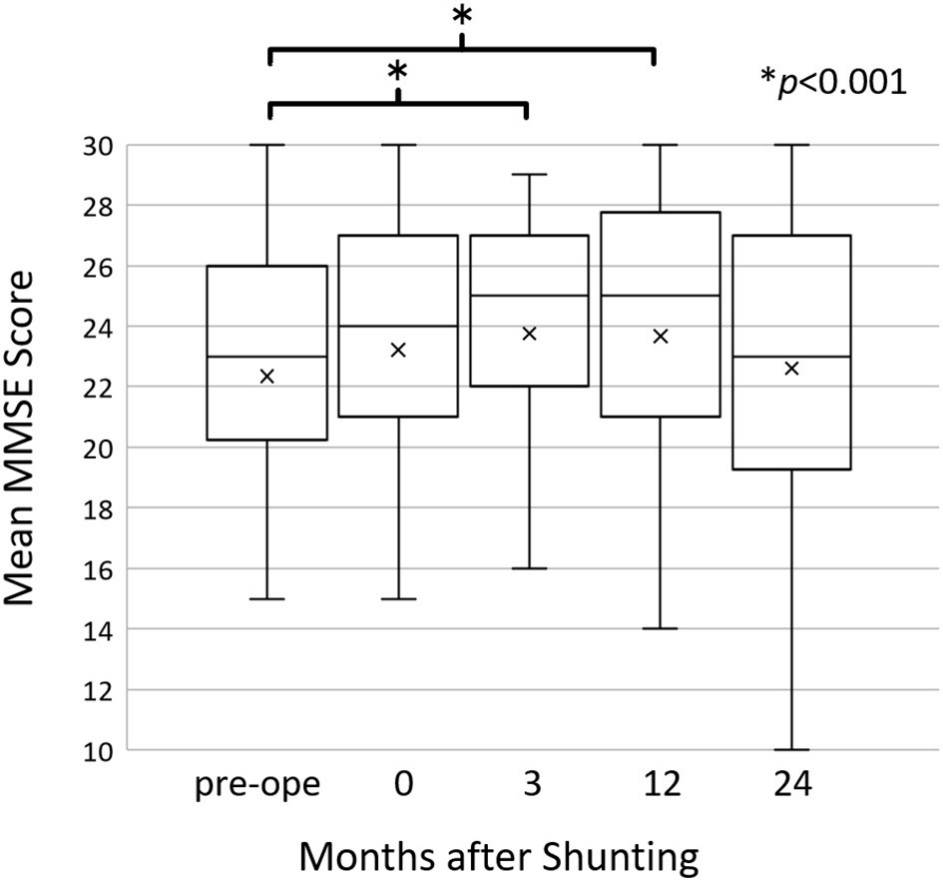
The mean Mini-Mental State Examination (MMSE) scores before and after shunting. The mean MMSE scores improved at 3 (*p* = 0.0002) and 12 (*p* = 0.004) months post-operatively compared to the preoperative scores; however, they returned to the preoperative level at 24 months post-operatively. Changes in the MMSE score were assessed using Wilcoxon's signed-rank test.

There was a significant improvement in the MMSE score from 22.4 ± 5.5 preoperatively to 23.8 ± 5.0 (*p* = 0.0002) and 23.7 ± 4.8 (*p* = 0.004) at 3 months and 1 year post-operatively, respectively. At 2 years post-operatively, the MMSE score was maintained at the preoperative level (MMSE = 22.6 ± 5.3) (Figure 1).

**Figure 2:**
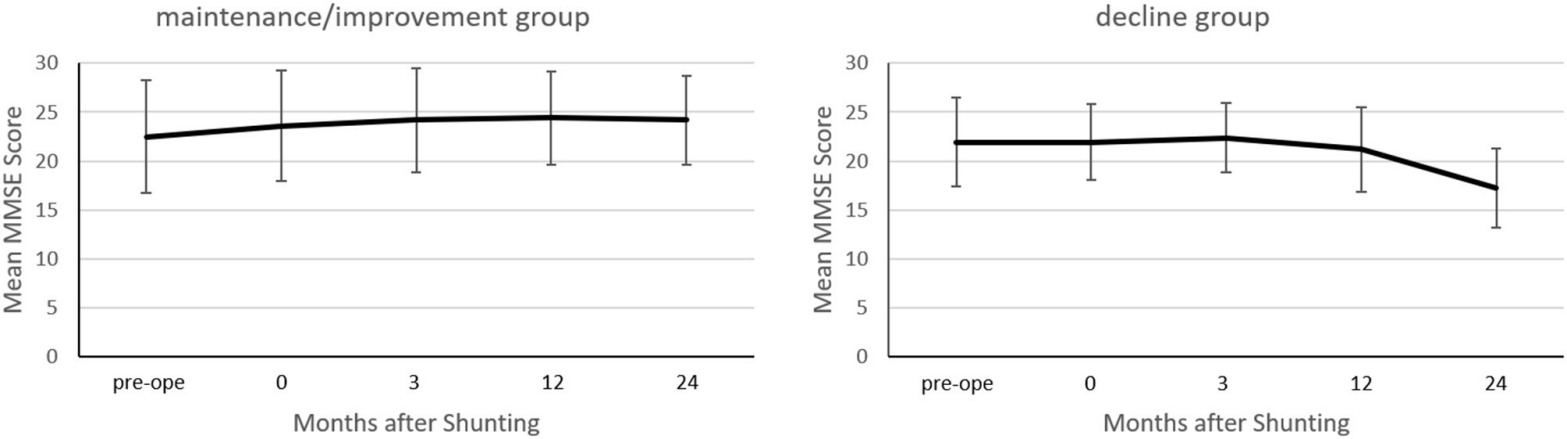
Between-group comparison of the mean Mini-Mental State Examination (MMSE) score before and after shunting. In the maintenance/improvement group, the mean MMSE score improved at 24 months post-operatively compared to preoperatively (*p* < 0.0001). Conversely, in the decline group, the mean MMSE score declined at 24 months post-/operatively compared to preoperatively (*p* = 0.0002). Changes in the MMSE score were assessed using Wilcoxon's signed-rank test.

At 2 years post-operatively, the MMSE scores increased by ≥2 points in 12 patients, decreased by ≥2 points in 11 patients, and showed almost no change in 25 patients. The maintenance/improvement group's MMSE score improved from 22.5 ± 5.7 to 24.2 ± 4.7 (*p* < 0.0001) (Figure 2). Conversely, the decline group's MMSE scores decreased from 21.9 ± 4.6 to 17.3 ± 4.0 (*p* = 0.0002).

### Cognitive Prognostic Factors

**Figure 3:**
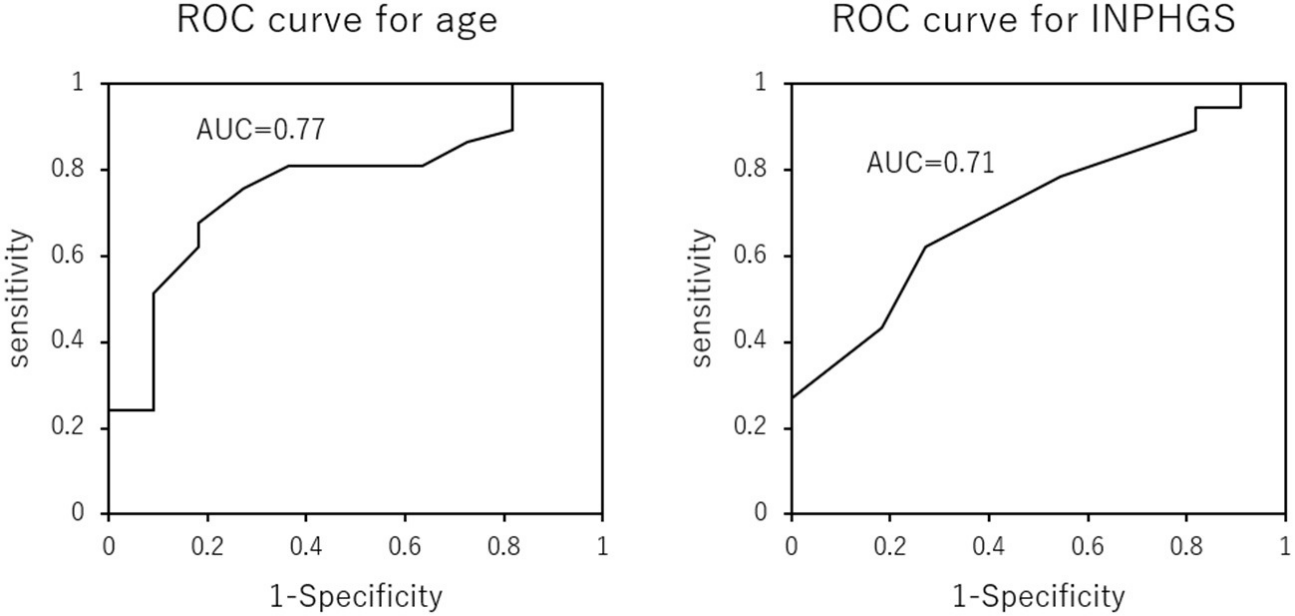
Receiver operating characteristic curves for the effect of age and Idiopathic Normal Pressure Hydrocephalus Grading Scale score on long-term cognitive prognosis after shunting. Older age and more severe symptoms predicted a poor cognitive prognosis 2 years after surgery.

There were 37 (77%) and 11 (23%) patients in the maintenance/improvement and decline groups, respectively. Student's *t-*test revealed significant between-group differences in age (*p* = 0.009) and INPHGS (*p* = 0.035) (Table 2). However, there were no significant between-group differences in time to surgery (*p* = 0.863), sex (*p* = 0.804), and surgical technique (*p* = 0.956). Logistic regression analysis revealed that the cutoffs for age and INPHGS score were 78 years (area under the curve = 0.77) and 5 points (area under the curve = 0.71), respectively (Figure 3). Receiver operating characteristic curve analysis revealed that poor prognosis was associated with old age and preoperative symptom severity.

**Table 2:** Between-group comparisons of the clinical data.

	**Maintenance/** **improvement** ** group** ** (*n =* 37)**	**Decline** ** group** ** (*n =* 11)**	***p-*value**
Age, mean (SD)	75.7 (±5.8)	80.8 (±3.7)	0.009^*^
Sex, % *(n)*			
Men	59.5% (22)	63.6% (7)	0.804
Women	40.5% (15)	36.3% (4)	
INPHGS (preoperatively), mean (SD)	4.9 (±2.2)	6.5 (±2.1)	0.035^*^
Days until surgery, mean (SD)	63.3 (±33.0)	61.6 (±17.1)	0.863
Type of surgery, % *(n)*			
LP shunt	81.1% (30)	81.8% (9)	0.956
VP shunt	18.9% (7)	18.2% (2)	

*INPHGS, Idiopathic Normal Pressure Hydrocephalus Grading Scale; LP, Lumboperitoneal; VP, Ventriculoperitoneal; SD, Standard deviation*.

* *p-value was calculated using Pearson's χ^2^ test for categorical variables and Student's t-test for continuous variables with normal distribution*.

**Figure 4:**
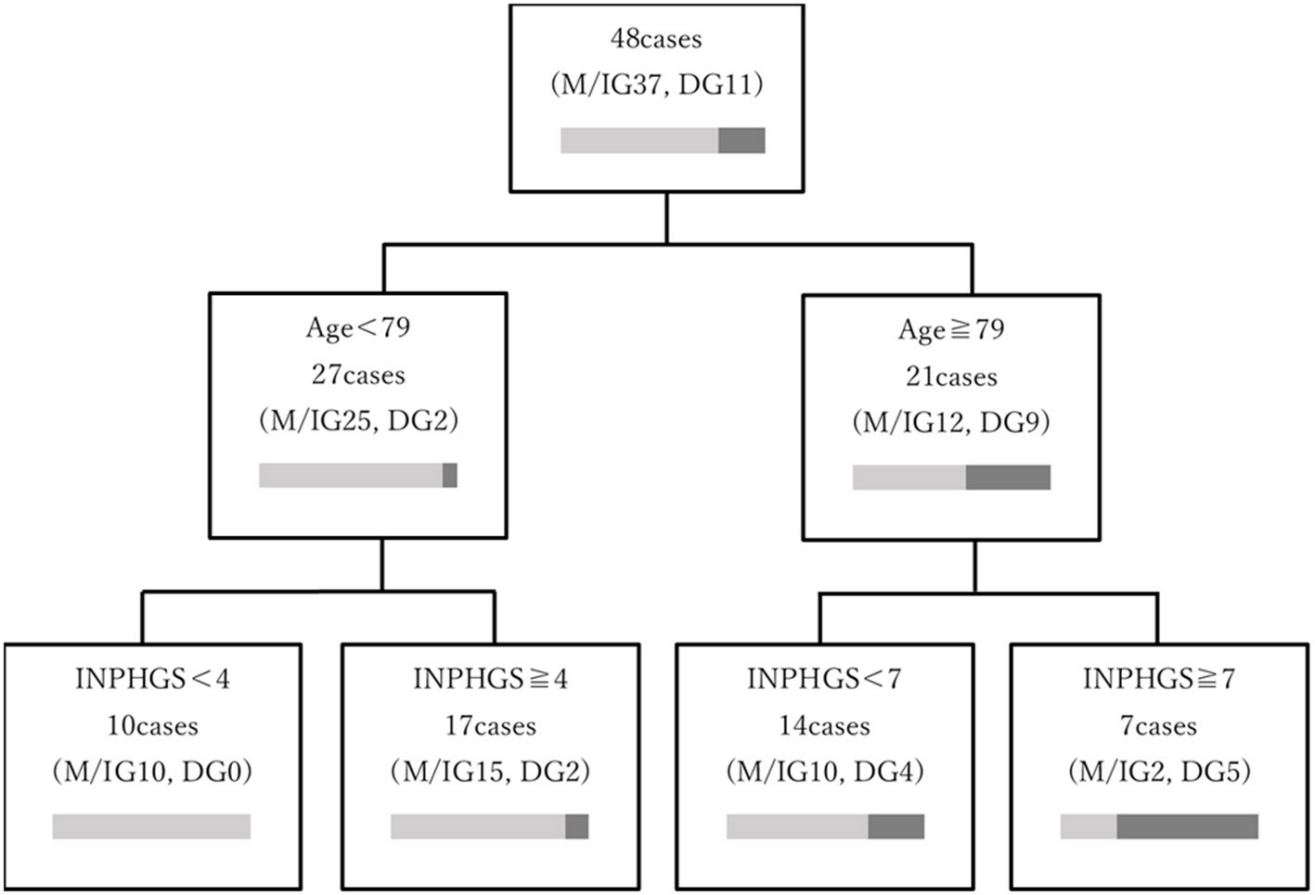
Decision tree model depicting the prognostic factors associated with long-term cognitive function after surgery for idiopathic normal pressure hydrocephalus. The decision tree was based on age and Idiopathic Normal Pressure Hydrocephalus Grading Scale (INPHGS) score as variables related to long-term cognitive function and ordered by the relative importance of each feature included in the model. The characteristics associated with the most favorable outcome (100% probability of maintenance/improvement) were age <79 years and INPHGS score <4 points. Furthermore, the combination of preoperative age ≥79 years with INPHGS score ≥7 showed the worst outcomes (29% probability of maintenance/improvement).

In a decision tree analysis for increasing the predictability of maintenance/improvement, age and INPHGS score were selected as the first and second nodes, respectively (Figure 4). Here, a preoperative age of <79 years and INPHGS score of <4 points had the best outcome (100% probability of maintenance/improvement) while a preoperative age of ≥79 years and INPHGS score of ≥7 had the worst outcome (29% probability of maintenance/improvement).

## Discussion

In this study, 77% of the patients with iNPH showed improved and maintained cognitive function after CSF shunting for at least 2 years post-operatively. This is the first study to perform a systematic evaluation of cognitive function for >2 years. Previous studies have reported that the MMSE scores of patients with iNPH improved by about 1 point within 1 year; however, it remained unclear whether this recovery was subsequently maintained (Solana et al., 2012; Shaw et al., 2016). Despite examining a small number of patients, some studies have reported no long-term improvement in cognitive function after shunting (Mori, 2001; Savolainen et al., 2002; Kahlon et al., 2007; McGirt et al., 2008). We found that the MMSE score improved by an average of 1.2 points at 1 year post-operatively and nearly returned to preoperative levels at 2 years post-operatively. Specifically, this indicates that cognitive function is maintained for 2 years.

Generally, iNPH is a progressive disease. Patients with untreated iNPH showed a decrease in the MMSE score by 3 points at 13 months after the diagnosis of iNPH (Andrén et al., 2014). The maintenance of cognitive function for >2 years indicates that CSF shunting has a significant therapeutic effect on cognitive function in patients with iNPH.

Although long-term cognitive prognosis varies widely across individuals, the predictive factors include age (*p* = 0.009) and INPHGS (*p* = 0.035). Future studies should determine the prediction accuracy.

This study included a relatively large number of older patients, with a mean age of 75.7 ± 5.8 and 80.8 ± 3.7 years in the maintenance/improvement and decline groups, respectively. Although the etiology of iNPH remains unclear, aging is considered as its greatest risk factor. More than 50% of patients with iNPH develop other neurodegenerative diseases. Therefore, age could be associated with long-term poor prognosis of cognitive function, since older age increases the probability of comorbidities (Golomb et al., 2000; Leal et al., 2018; Libard et al., 2018). In the short term, comorbid AD does not affect the post-operative improvement of iNPH symptoms; however, there have been no long-term follow-up studies (Bech-Azeddine et al., 2007).

In this study, six patients had a history of AD, and one patient developed AD during the follow-up period. Of them, one patient showed an improved MMSE score after the shunting; however, there was a gradual cognitive decline after 1 year. Further, the patient was diagnosed with post-operative AD based on symptoms and a single-photon emission computed tomography scan showing hypovolemia in the bilateral posterior cingulate gyri. After 2 years, the patients diagnosed with AD had a greater decrease in the MMSE score (by 11 and 5 points, respectively) than those without AD. In addition to AD, cerebral vascular disease has been reported to be highly comorbid with iNPH (Bech-Azeddine et al., 2007). None of the participants experienced cerebral infarction within 2 years of the surgery. Two of the six patients with a history of cerebral infarction had a decrease MMSE score (by 11 and 3 points, respectively). However, all patients did not undergo close examination for dementia complications, including AD, during the follow-up period. Therefore, there is a need to detect the involvement of other diseases for appropriate iNPH treatment.

Regarding the comorbidities of iNPH, CSF circulation in the glymphatic pathway has recently been shown to be associated with amyloid production in the brain (Mortensen et al., 2019). CSF amyloid and p-tau levels are reportedly increased post-operatively in patients with iNPH (Moriya et al., 2015). Consequently, patients with iNPH may have progressive amyloid and p-tau accumulation resulting from impaired CSF circulation, which may increase the incidence of comorbidity, including AD (Reeves et al., 2020). Our finding that as many as 77% of patients showed improved and maintained cognitive function for 2 years after surgery could be indicative of inhibition of the pathophysiology of dementia itself.

This study shows that younger age and less severe iNPH contribute to a favorable long-term prognosis of cognitive function. This indicates the importance of early diagnosis and treatment for the long-term maintenance of cognitive function. Moreover, Andrén et al. (2014) reported that iNPH symptom progression is irreversible, and that delaying surgery could worsen symptoms. This indicates the need for early diagnosis and treatment to improve long-term cognitive prognosis in patients with iNPH.

This study has several limitations. First, the duration of this study was short, and future studies with longer follow-up periods are needed to validate our findings. We are currently designing a more than 3-year follow-up study that will include the participants of this study. Second, the MMSE, a screening test that can assess overall cognitive function, has limited utility in assessing cognitive function in iNPH, especially when used alone. In this study, the cognitive function of most patients is at the level of mild cognitive impairment. A cognitive function test suitable for mild cognitive impairment, such as the Montreal Cognitive Assessment, is considered to have better accuracy (Nasreddine et al., 2005). We should use multiple tests to assess a wide range of cognitive functions. Finally, although we incorporated multiple predictors, not all potentially relevant predictors were incorporated. For example, future studies should consider including iNPH-specific neuroimaging parameters (e.g., Evans index) and factors associated with AD and atherosclerosis. However, for comorbidities, the diagnosis of AD and other comorbidities may require pathological examination, which was ethically difficult to clarify in all cases because of the need for invasive procedures. In conclusion, patients with iNPH showed significantly improved cognitive function for 3 months to 1 year after CSF shunt surgery, which was maintained until at least 2 years. Cognitive function improvement and maintenance were more pronounced in younger patients with milder disease. Therefore, early diagnosis and prompt treatment are important for long-term improvement and maintenance of cognitive function in patients with iNPH.

## Data Availability Statement

The original contributions presented in the study are included in the article/Supplementary Materials, further inquiries can be directed to the corresponding author/s.

## Ethics Statement

The studies involving human participants were reviewed and approved by the ethics committee of Osaka Medical College. Written informed consent for participation was not required for this study in accordance with the national legislation and the institutional requirements.

## Author Contributions

AK, YK, and MW made substantial contributions to the conception and design of the study. AK, YK, KK, ST, and RS collected data regarding the participants and task performance. AK, YK, RY, NI, MF, NN, SK, and TK analyzed the data. AK and YK wrote the manuscript. RS and MW supervised this project. All authors read and approved the submitted version.

## Conflict of Interest

The authors declare that the research was conducted in the absence of any commercial or financial relationships that could be construed as a potential conflict of interest.
